# The Relationship Among Morningness-Eveningness, Sleep Duration, Social Jetlag, and Body Mass Index in Asian Patients With Prediabetes

**DOI:** 10.3389/fendo.2018.00435

**Published:** 2018-08-15

**Authors:** Thunyarat Anothaisintawee, Dumrongrat Lertrattananon, Sangsulee Thamakaison, Ammarin Thakkinstian, Sirimon Reutrakul

**Affiliations:** ^1^Department of Family Medicine, Faculty of Medicine Ramathibodi Hospital, Mahidol University, Bangkok, Thailand; ^2^Section for Clinical Epidemiology and Biostatistics, Faculty of Medicine Ramathibodi Hospital, Mahidol University, Bangkok, Thailand; ^3^Division of Endocrinology and Metabolism, Department of Medicine, Faculty of Medicine Ramathibodi Hospital, Mahidol University, Bangkok, Thailand; ^4^Division of Endocrinology, Diabetes and Metabolism, Department of Medicine, University of Illinois at Chicago, Chicago, IL, United States

**Keywords:** prediabetes, circadian, body mass index, eveningness, sleep duration, social jetlag

## Abstract

**Background:** Circadian system is known to influence energy metabolism. Recent evidence suggested that evening preference could be associated with higher body mass index (BMI). Moreover, evening preference is known to be associated with insufficient sleep duration and greater social jetlag, both described to be associated with obesity. This study aimed to explore whether morningness-eveningness was directly associated with BMI or its effect was transmitted through sleep duration or social jetlag in patients with prediabetes.

**Methods:** A total 2,133 patients with prediabetes were enrolled. Morningness-eveningness was assessed using a Composite Scale of Morningness (CSM). Average weekly sleep duration and sleep timing were obtained, and social jetlag was calculated. BMI was calculated by weight (kg)/height^2^ (m^2^). A mediation analysis was performed based on two pathways, i.e. CSM→sleep→duration→BMI and CSM→social jetlag→BMI. A sequential equation model was used to estimate the direct and indirect effects of CSM on BMI.

**Results:** Mean (SD) age and BMI were 63.6 (9.2) years and 25.8 (4.0) kg/m^2^. For CSM→sleep duration→BMI pathway, every one point decrease in CSM (more evening preference) was associated with a decrease in sleep duration by 0.054 h (95% CI 0.043–0.066), whereas sleep duration was negatively associated with BMI (coefficient = −0.156, 95%CI −0.288, −0.024). Mediation analysis indicated that a change in CSM (from 90th to 10th percentile, more evening preference) was associated with a decrease in sleep duration and an increase in BMI by 0.102 kg/m^2^ (95% CI 0.015, 0.207). In addition, this change in CSM was directly associated with an increase in BMI by 0.511 kg/m^2^ (95%CI 0.030, 0.952). The CSM→social jetlag→BMI pathway analysis revealed that social jetlag was not significantly associated with BMI. A subgroup analysis in those aged ≤60 years (*n* = 784) revealed that each hour increase in social jetlag was associated with an increase in BMI by 0.56 kg/m^2^ (*p* = 0.026) while CSM and sleep duration were not.

**Conclusion:** In patients with prediabetes, more evening preference was directly associated with higher BMI and indirectly through insufficient sleep duration, while social jetlag did not mediate the relationship between CSM and BMI. In those ≤60 years, only greater social jetlag was associated with higher BMI. These data could inform further interventional studies to reduce BMI in this high risk group.

## Introduction

Diabetes is a global health problem. In the United States, 9.4% of the population, or 30.3 million people, were estimated to have diabetes in 2015 (1). The world's heaviest burden of diabetes, however, is in the Western Pacific Region, with 159 million people having diabetes in 2017, with the number expected to rise by 15% by 2045 (2). Prediabetes, a condition in which blood glucose levels are elevated but do not yet meet a criteria for diabetes, is a precursor which markedly increases the risk of developing type 2 diabetes and cardiovascular disease (1). Diabetes prevention with intensive lifestyle interventions has been shown to significantly reduce the risk of diabetes progression (3). In the Diabetes Prevention Program, exercise and weight loss of 7% in patients with prediabetes resulted in a 58% reduction in the risk of developing diabetes, confirming the crucial role of adiposity in abnormal glucose metabolism (3). Therefore, identifying novel factors influencing adiposity in prediabetes patients could lead to interventions to prevent diabetes in this high risk group.

The circadian system, controlled by the master circadian clock located in the suprachiasmatic nuclei of the hypothalamus, is known to play a major role in regulating daily rhythms of metabolism, sleep/wake cycle, feeding behavior, and hormonal secretions (4). There is evidence that circadian disruption or circadian misalignment has detrimental effects on energy metabolism. Experiments utilizing forced-desynchrony protocols which the participants ate and slept on a recurring 28-h day have been shown to result in reduced resting metabolic rate and leptin levels (5, 6). Night shift work, often associated with chronic circadian misalignment, has been shown to be a risk factor for developing obesity (7). This could be partially due to insufficient and/or poor-quality sleep, well-known risk factors for obesity (8, 9) often observed in shift workers. In addition, alterations in meal timing itself can affect circadian regulation (10). In non-shift working population, milder forms of circadian misalignment can be observed, such as in those with evening preference.

The time of day during which individuals prefer to sleep or perform daily activities denotes morningness-eveningness or chronotype. Individuals with evening preference, typically with a later bedtime than those with morning preference, often have a greater degree of circadian misalignment between behavioral rhythms and the endogenous central circadian clock (11). More evening preference has been shown to be associated with greater social jetlag, which is a phenomenon resulting from shifting sleep timing between work days and free days resembling traveling across time zones (12). Emerging evidence from studies in adolescents as well as in general population suggested that evening preference (or late bedtime) and social jetlag were associated with increased adiposity (13–15), although some found this relationship in overweight individuals only (14). Whether evening preference is associated with overweight/obesity in patients with prediabetes, a group at high risk for developing diabetes, has not been previously explored.

Therefore, this study aimed to explore the contribution of morningness-eveningness preference to body mass index (BMI) of patients with prediabetes. By employing mediation analyses, we further examined if this association is mediated through factors known to be associated with both eveningness and obesity, including insufficient sleep duration and social jetlag, or whether there is a direct contribution of morningness-eveningness preference to BMI.

## Materials and methods

This cross-sectional study utilized the baseline data from the cohort study of prediabetes patients, which has been conducted since October 2014 at the outpatient clinic of Department of Family Medicine, Ramathibodi Hospital, Bangkok, Thailand. Prediabetes patients aged ≥18 years were recruited. Criteria used for diagnosis prediabetes were fasting plasma glucose (FPG) between 100 and 125 mg/dl (5.6–6.9 mmol/L) or hemoglobin A1c (HbA1c) between 5.70 and 6.49% (38.80–47.44 mmol/mol) (16). Patients were excluded if they had any of following: FPG ≥126 mg/dl (≥7.0 mmol/L), HbA1c level ≥6.5% (48.0 mmol/mol), or were shift workers. The study's protocol was approved by the Ethical clearance Committee of Ramathibodi Hospital, Mahidol University. All participants signed written informed consent.

### Data collection

Demographic data (i.e., age, sex, educational level), family history of diabetes mellitus in first degree relatives, history of smoking (never or current/past) and alcohol consumption (never or current/past) were collected by trained interviewers. Depressive symptoms, previously shown to be related to overweight/obesity (17), were assessed using the Thai version of the Center for Epidemiologic Studies-Depression (CESD) Scale (18). Underlying diseases (i.e., hypertension, dyslipidemia, and chronic kidney disease defined as estimated glomerular filtration rate <60 ml/min/1.73 m^2^) and date of diagnosis of prediabetes were reviewed from patient's medical records by investigating physicians (TA, ST, and DL). Height and weight were measured with a digital scale (Seca 284, CA, U.S.A., precision to 0.1 cm and 0.1 kg) at the date of enrollment by trained staff. BMI was calculated by weight (kg)/height^2^ (m^2^).

### Morningness-eveningness assessment

Morningness-eveningness preference was assessed using the validated Thai version of the Composite Scale of Morningness (CSM) (19). The CSM consists of 13 questions regarding the preferred time individuals would like to wake up and go to bed, preferred time for physical and mental activity, and subjective alertness. The total score ranges from 13 (i.e., extreme eveningness) to 55 (i.e., extreme morningness).

### Subjective sleep and social jetlag assessment

Participants were interviewed to collect the data of sleep characteristics including sleep duration and sleep quality, as well as social jetlag. Sleep duration was obtained by the question of “During the past month, how many hours of actual sleep did you get at night?” This question was asked separately for weekdays and weekends. Average sleep duration was then calculated as [(sleep duration on weekdays^*^5) + (sleep duration on weekend^*^2)]/7.

To assess sleep quality over the previous month, we utilized the Pittsburgh Sleep Quality Index (PSQI) score, also validated in Thai (20). A modified PSQI score was created by removing the sleep duration component to assess sleep quality independently from sleep quantity (21). Higher scores reflect poorer sleep quality.

The participants were also asked about their usual bedtime, wake-up time, and sleep onset latency on weekdays and weekends over previous month. Mid sleep time of weekdays and weekends were estimated from these information as the midpoint between sleep onset and wake time. Social jetlag (in hours) was then calculated by the absolute difference between mid-sleep time on weekdays and weekends.

### Dietary assessment

Information about all food and beverages intake in the past 24 h before the date of interview were collected by 24-h food recall. Time of the day (presented in 24-h clock time), types and portion of food intake including beverages and snack of each meal were collected. Total daily calorie intake was calculated by dieticians using a Thai food database (INMUCAL-Nutrients V.3, Institute of Nutrition, Mahidol University, Bangkok, Thailand).

### Physical activity assessment

The physical activity of study's participants was assessed by Global Physical Activity Questionnaire (GPAQ) version 2 (22). This questionnaire asked about the time that participants spent for vigorous- and moderate-intensity activities according to work, travel to, and from places, and recreational activities. The intensity of physical activities was measured as Metabolic Equivalents (METs) that one MET was equivalent to a caloric consumption of 1 kcal/kg/h, and four and eight METS were assigned to the time spent in moderate and vigorous activities, respectively. Total physical activity for each participant was then calculated by summation of MET values of work, travel to and from places, and recreational activities.

### Statistical analysis

Characteristics of the participants were presented as mean (standard deviation, SD) or median (range) for continuous data and as frequency and percentage for categorical data. Univariate linear regression analysis was applied to assess the association between study factor (i.e., CSM score) and mediators (i.e., sleep duration and social jetlag) and BMI outcome. In addition, associations between study factor, mediators, BMI, and other covariables, including demographic variables (i.e., age, sex, educational level), risk behavior (i.e., smoking and alcohol use), depressive symptoms, modified PSQI, dietary parameters (i.e., breakfast and dinner time, and total daily calorie) and physical activity were assessed.

Mediation analysis for continuous data was then applied to assess the direct effect of morningness-eveningness preference (CSM score) on BMI and indirect effects of CSM score on BMI mediated by sleep duration and social jetlag.

**Figure 1:**
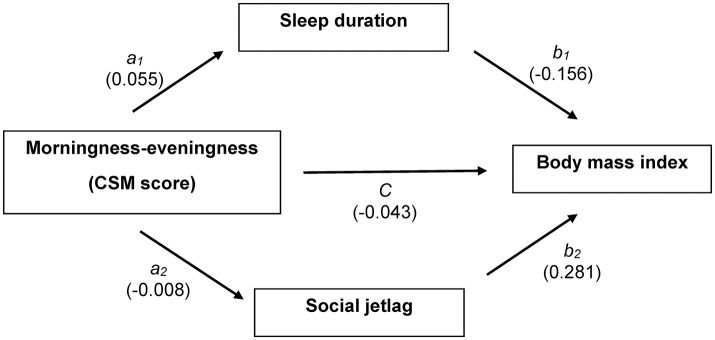
Causal association diagram between CSM score and body mass index with multiple mediation models; CSM score is an independent variable, sleep duration and social jetlag are mediators, and body mass index is an outcome. Coefficient for each path is shown.

Causal pathways among CSM score, sleep duration, social jetlag, and BMI were constructed as illustrated in Figure 1. According to these pathways, three equations were constructed as follows.

Mediation model for sleep duration: path *a*_1_

$$\begin{array}{r} Sleepduration_{i}=a_{0}+a_{1}CSM_{i}+\underset{k}{\sum }e_{k}z_{k} \end{array}$$

Mediation model for social jetlag: path *a*_2_

$$\begin{array}{r} Socialjetlag_{i}=b_{0}+a2CSM_{i}+\underset{k}{\sum }e_{k}z_{k} \end{array}$$

Outcome model for BMI: paths *b*_1_, *b*_2_, and *c*′

$$\begin{array}{l} BMI_{i}=c_{0}+b_{1j}Sleepduration_{j}+b_{2j}Socialjetlag_{j}+c^{\prime }CSM_{i}\\ +\underset{l}{\sum }e_{k}z_{k}\\ \;z_{k}=confounders \end{array}$$

Sleep duration was regressed on CSM score (*a*_1_) as shown in Equation (1). Social jetlag was then regressed on CSM score (*a*_2_) as shown in Equation (2). Finally, BMI was fitted on sleep duration mediator (path *b*_1_), social jetlag mediator (path *b*_2_), and CSM score as direct effect (path *c*) as shown in Equation (3). The three equations were adjusted for confounding factors, which were significantly associated with each equation including age, sex, educational level, smoking, and alcohol use, modified PSQI score, CESD score, dinner, and breakfast time. Product coefficient method was applied to estimate the average causal mediation effect (ACME) of sleep duration (*a*_1_*b*_1_) and social jetlag (*a*_2_*b*_2_).

In addition, two sensitivity analyses were performed. Because dietary recall is subjected to bias especially with regards to caloric intake, we performed analyses excluding caloric consumption data. We also analyzed the participants according to age since social jetlag is known to be more prevalent in younger age (14). Since our population's mean age was 63.6 years, we divided them into >60 years or ≤60 years according to Thai's definition of elderly (23).

A bootstrap analysis with 1,000 replications was used to estimate ACMEs and their 95% confidence interval (CI) using bias-corrected bootstrap technique. All analyses were performed using STATA version 14. *P* < 0.05 was considered as significant level for all tests.

## Results

A total of 2,133 prediabetes patients were eligible for this study. Baseline characteristics of participants are presented in Table 1. Means (SD) age and BMI of participants were 63.6 (9.2) years and 25.8 (4.0) kg/m^2^. Percentages of female, current/past smoker, and alcohol user were 65.7, 24.6, and 47.3%, respectively. Around 40% of participants had family history of diabetes in first degree relatives. Most participants had dyslipidemia (88.2%) and hypertension (68.4%) but only 4.9% had chronic kidney disease. Mean (SD) of CSM (morningness-eveningness preference) score was 46.6 (4.8). Average sleep duration was 7.0 (1.3) h and modified PSQI scores were 6.9 (2.0). Medians and ranges of social jetlag and CESD score were 0 (0–4) h and 6 (0–46). Dietary recalls revealed that means breakfast and dinner times were 08:03 (1:06) and 18:00 (1:13), respectively, and total daily calorie intake was 1,027.9 (410.8). Physical activity was 128.57 MET (0–4,080).

**Table 1:** Demographic data, morningness-eveningness, sleep characteristics, and dietary parameters (*n* = 2,133).

**Factor**	**Result (SD; range)**
**DEMOGRAPHICS**	
Age (years)	63.6 (9.2; 32–92)
Female	1,401 (65.7)
Body mass index (kg/m^2^)	25.8 (4.0; 12.36–54.96)
Educational level	
Primary or less	738 (34.6)
Secondary school	607 (28.4)
College or higher	788 (37)
**SMOKING HISTORY**	
Never	1,608 (75.4)
Current/past	525 (24.6)
**ALCOHOL USE**	
Never	1,124 (52.7)
Current/past	1,009 (47.3)
Family history of diabetes	832 (39.0)
Hypertension	1,457 (68.4)
Dyslipidemia	1,877 (88.2)
Chronic kidney disease	105 (4.9)
CESD score*	6 (4.91; 0–46)
Physical activity*	128.57 (269.76; 0–4,080)
**MORNINGNESS-EVENINGNESS, SOCIAL JETLAG, AND SLEEP CHARACTERISTICS**	
Composite scale of morningness (CSM) score	46.6 (4.8; 21–55)
Social jetlag* (h)	0 (0.46; 0–4)
**SUBJECTIVE SLEEP ASSESSMENT**	
Sleep duration (h)	7.0 (1.3; 1–11)
Modified PSQI	6.9 (2.0; 3–14)
**DIETARY PARAMETERS**	
Breakfast time (hh:min)	08:03 (01:06; 03:00–12:00)
Dinner time (hh:min)	18:00 (01:13; 12:30–00:00)
Total daily calories	1,027.9 (410.8; 172–3,918)

CESD, Center for Epidemiologic Studies-Depression; PSQI, Pittsburgh sleep quality index. *Median.

### Morningness-eveningness, mediators (sleep duration, and social jetlag) and BMI

Univariate regression analyses were performed (see Table 2). These revealed that more evening preference (lower CSM score), greater social jetlag and shorter sleep duration were associated with higher BMI. In addition, younger age, being female, and lower educational level were associated with higher BMI. Higher physical activity was associated with lower BMI, but the result was not statistically significant. Total daily calories, but not meal timing, were associated with BMI.

**Table 2:** Univariate regression analysis between morningness-eveningness, sleep characteristics, demographic data, and body mass index.

**Variables**	**BMI**		**Sleep duration**		**Social jetlag**	
	**Coefficient**	***p*-value**	**Coefficient**	***p*-value**	**Coefficient**	***p*-value**
**MORNINGNESS-EVENINGNESS, SOCIAL JETLAG, AND SLEEP CHARACTERISTICS**						
CSM score	−0.080	<0.001	0.053	<0.001	−0.011	<0.001
Social jetlag	0.429	0.008	0.037	0.491	–	–
Sleep duration	−0.239	<0.001	–	–		
Modified PSQI	0.055	0.168	0.027	0.040	−0.007	0.158
**DEMOGRAPHICS**						
Age	−0.076	<0.001	0.009	0.001	−0.010	<0.001
Male	−0.392	0.014	0.241	<0.001	0.018	0.327
BMI	–	–	−0.239	<0.001	0.429	0.008
Educational level	−0.316	0.045	−0.011	0.836	0.069	<0.001
Smoking	−0.236	0.180	0.213	<0.001	0.045	0.031
Alcohol use	0.085	0.576	0.181	<0.001	0.032	0.072
CESD	0.007	0.628	−0.022	<0.001	0.0004	0.798
Physical activity	−0.0005	0.096	9.67*e*−06	0.916	−0.00002	0.529
**DIETARY PARAMETERS**						
Breakfast time	0.054	0.419	0.052	0.017	0.008	0.300
Dinner time	−0.092	0.129	−0.055	0.007	0.022	0.002
Total daily calories	−0.0005	0.004	0.0002	0.877	0.00006	0.004

*CESD, Center for Epidemiologic Studies-Depression; CSM, composite scale of morningness; PSQI, Pittsburgh sleep quality index*.

For sleep duration, the results revealed that more evening preference, along with lower modified PSQI score, younger age, being female, non-smoking, non-alcohol use and greater depressive symptoms were significantly associated with shorter sleep duration. In addition, earlier breakfast time and later dinner time were also associated with shorter sleep duration.

For social jetlag, more evening preference (lower CSM score) was significantly associated with greater social jetlag. In addition, younger age, higher educational level, smoking, and later dinner time were also associated with a greater amount of social jetlag.

### Mediation analysis

Because more evening preference was associated with both mediators (i.e., shorter sleep duration and greater social jetlag) and higher BMI, and both mediators (i.e., shorter sleep duration and greater social jetlag) were associated with higher BMI, we further explored if the association between CSM score and BMI was mediated by sleep duration and/or social jetlag. Two mediation equations for sleep duration and social jetlag and one outcome equation for BMI were constructed, adjusting for age, sex, smoking, alcohol use, educational level, modified PSQI, CESD, breakfast time, dinner time, and total daily calories. Their coefficients adjusted for confounding factors are illustrated in Table 3.

**Table 3:** Multiple mediation analysis of CSM score and body mass index.

**Equations**	**Factor**	**Coefficient**	**SE**	***Z***	***P***	**95% CI**
Sleep duration	CSM score	0.055	0.006	9.20	<0.001	0.043, 0.066
	Age	0.001	0.003	0.46	0.644	−0.005, 0.008
	Male	0.145	0.081	1.79	0.074	−0.014, 0.303
	Education	0.019	0.057	0.32	0.746	−0.094, 0.131
	Smoking	0.066	0.087	0.76	0.449	−0.105, 0.236
	Alcohol use	0.120	0.065	1.86	0.063	−0.007, 0.248
	CESD score	−0.015	0.006	−2.50	0.012	−0.026, −0.003
	Physical activity	−0.0001	0.0001	−0.98	0.327	−0.0003, 0.0001
	Modified PSQI	0.040	0.014	2.77	0.006	0.012, 0.069
	Breakfast time	0.084	0.024	3.50	<0.001	0.037, 0.131
	Dinner time	−0.038	0.021	−1.77	0.076	−0.080, 0.004
	Total daily calories	−0.00003	0.0001	−0.40	0.690	−0.0002, 0.0001
Social jetlag	CSM score	−0.008	0.002	−3.74	<0.001	−0.012, −0.004
	Age	−0.009	0.001	−8.14	<0.001	−0.011, −0.007
	Male	−0.0004	0.029	0.01	0.990	−0.057, 0.056
	Education	0.047	0.021	2.28	0.023	0.007, 0.087
	Smoking	0.024	0.031	0.77	0.439	−0.037, 0.086
	Alcohol use	0.010	0.023	0.45	0.654	−0.035, 0.056
	CESD score	−0.0001	0.002	−0.06	0.952	−0.004, 0.004
	Physical activity	−0.00002	0.00003	−0.58	0.565	−0.0001, 0.00005
	Modified PSQI	−0.005	0.005	−0.96	0.335	−0.015, 0.005
	Breakfast time	0.012	0.009	1.33	0.182	−0.005, 0.028
	Dinner time	0.009	0.008	1.10	0.272	-0.007, 0.024
	Total daily calories	0.00003	0.00002	1.10	0.271	−0.00002, 0.00007
BMI outcome	Social jetlag	0.281	0.187	1.51	0.132	−0.085, 0.647
	Sleep duration	−0.156	0.067	−2.31	0.021	−0.288, −0.024
	CSM score	−0.043	0.019	−2.26	0.024	−0.079, −0.006
	Age	−0.076	0.010	−7.68	<0.001	−0.095, −0.056
	Male	−0.181	0.252	−0.72	0.473	−0.674, 0.313
	Education	−0.555	0.179	−3.10	0.002	−0.905, −0.204
	Smoking	0.065	0.271	0.24	0.810	−0.466, 0.596
	Alcohol use	0.278	0.202	1.38	0.168	−0.117, 0.674
	CESD score	−0.028	0.019	−1.49	0.135	−0.064, 0.009
	Physical activity	−0.0005	0.0003	−1.46	0.146	−0.001, 0.0002
	Modified PSQI	0.070	0.045	1.56	0.120	−0.018, 0.158
	Breakfast time	0.003	0.075	0.04	0.966	−0.143, 0.149
	Dinner time	−0.162	0.067	−2.42	0.016	−0.293, −0.030
	Total daily calories	−0.0005	0.0002	−2.55	0.011	−0.0009, −0.0001

The results from the mediation equation of sleep duration showed that every one unit increase in CSM score (more morning preference) was significantly associated with an increase in sleep duration by 0.055 h (95% CI: 0.043, 0.066). For the equation of social jetlag mediator, the result suggested that increased CSM score (more morning preference) was significantly associated with decreased social jetlag with the coefficient of −0.008 (95% CI: −0.012, −0.004).

The results of the BMI outcome model (Table 3) revealed that increased sleep duration was significantly associated with decreased BMI, with a coefficient of −0.156 (95% CI: −0.288, −0.024). More morning preference (higher CSM score) was also significantly associated with lower BMI (*p* = 0.024). Social jetlag, however, was not significantly associated with BMI (*p* = 0.132).

A bootstrap with 1,000 replications was then applied to estimate the ACMEs of CSM score on BMI mediated by sleep duration (ACME_1_), social jetlag (ACME_2_) and to estimate the direct effect of CSM on BMI (*c*′), see Table 4. The results revealed the ACME_1_ was significant, i.e., that every one unit increase in CSM score (more morning preference) would be associated with an increase in sleep duration, which then significantly decreased BMI by 0.0085 kg/m^2^ (95% CI: 0.0167, 0.0003). However, the association between CSM score and BMI mediated by social jetlag was not significant (ACME_2_ = −0.0022, 95% CI: −0.0056, 0.0011). In addition, we found that there was also a direct effect of CSM on BMI such that every one unit increase in CSM score (more morning preference) was associated with a decrease in BMI by 0.043 kg/m^2^ (95% CI: −0.080, −0.005).

**Table 4:** Causal association effects between CSM score and body mass index.

**Parameter**	**Model**	**Pathway**	**Beta**	**95% CI**
ACME_1_	Sleep duration model	CSM→Sleep duration→BMI (a_1_b_1_)	−0.0085	−0.0167, −0.0003
ACME_2_	Social jetlag model	CSM→Social jetlag→BMI (a_2_b_2_)	−0.0022	−0.0056, 0.0011
Direct effect	BMI model	CSM→BMI (c′)	−0.043	−0.0797, −0.0054
Total effects			0.0533	0.0175, 0.0891
**PERCENTAGE OF DIRECT AND MEDIATION EFFECTS OF CSM ON BMI**				
Percent mediation effect through sleep duration			15.94	
Percent mediation effect through social jetlag			4.20	
Direct effect			79.86	

*ACME, average causal mediation effect*.

Additional analyses were performed excluding caloric intake from the models. The results were similar. Bootstrap analysis revealed that the ACME_1_ was significant, i.e., that every one unit increase in CSM score (more morning preference) would be associated with an increase in sleep duration, which then significantly decreased BMI by 0.008 kg/m^2^ (95% CI: −0.016, −0.0006). The association between CSM score and BMI mediated by social jetlag was not significant. There was also a direct effect of CSM on BMI such that every one unit increase in CSM score (more morning preference) was associated with a decrease in BMI by 0.042 kg/m^2^ (95% CI: −0.079, −0.004).

The distribution of CSM score was explored with 10th, 50th, and 90th percentile of 40, 47, and 52, respectively. A difference in CSM score between 90th and 10th percentile in our cohort (12 points, more evening preference) was associated with a decrease in sleep duration and an increase in BMI by 0.102 kg/m^2^ (95% CI 0.015, 0.207), and was directly associated with an increase in BMI by 0.511 kg/m^2^ (95%CI 0.030, 0.952). Percentages of mediation effects of sleep duration and social jetlag on BMI were 15.94 and 4.20%, respectively, while the direct effect by CSM contributed 79.86% (Table 4).

### Subgroup analysis by age (≤ 60 vs. >60 years)

Mediation analysis in participants aged ≤60 years is shown in Table 5. The BMI outcome model revealed that sleep duration and CSM score were not associated with BMI. However, greater social jetlag was significantly associated with BMI. Every 1 h increase in social jetlag was associated with higher BMI by 0.56 kg/m^2^ (95% CI: 0.06, 1.06).

**Table 5:** Multiple mediation analysis of CSM score and body mass index in participants age ≤60 years (*n* = 784).

**Equations**	**Factor**	**Coefficient**	**SE**	***Z***	***P***	**95% CI**
Sleep duration	CSM score	0.051	0.010	5.32	<0.001	0.032, 0.069
	Male	0.180	0.126	1.42	0.155	−0.068, 0.428
	Education	0.034	0.092	0.37	0.712	−0.146, 0.214
	Smoking	0.078	0.132	0.59	0.553	−0.180, 0.337
	Alcohol use	0.084	0.101	0.83	0.405	−0.114, 0.282
	CESD score	−0.014	0.008	−1.71	0.088	−0.031, 0.002
	Physical activity	0.00006	0.0001	0.40	0.688	−0.0002, 0.0004
	Modified PSQI	0.046	0.022	2.06	0.040	0.002, 0.090
	Breakfast time	0.121	0.037	3.25	0.001	0.048, 0.195
	Dinner time	−0.050	0.033	−1.52	0.129	-0.115, 0.015
	Total daily calories	−0.00009	0.0001	−0.83	0.407	−0.0003, 0.0001
Social jetlag	CSM score	−0.014	0.005	−3.15	0.002	−0.024, −0.005
	Male	0.007	0.061	0.12	0.905	-0.113, 0.127
	Education	0.141	0.044	3.17	0.002	0.054, 0.228
	Smoking	0.002	0.064	0.03	0.977	-0.123, 0.127
	Alcohol use	0.041	0.049	0.84	0.400	-0.055, 0.137
	CESD score	−0.001	0.004	−0.35	0.723	-0.010, 0.007
	Physical activity	−0.00002	0.00007	−0.33	0.742	−0.0002, 0.0001
	Modified PSQI	−0.015	0.011	−1.38	0.168	−0.036, 0.006
	Breakfast time	0.006	0.018	0.31	0.757	−0.030, 0.041
	Dinner time	0.026	0.016	1.65	0.099	−0.005, 0.058
	Total daily calories	0.00006	0.00005	1.23	0.220	−0.00004, 0.0002
BMI outcome	Social jetlag	0.556	0.255	2.18	0.029	0.056, 1.06
	Sleep duration	0.061	0.123	0.50	0.619	−0.181, 0.303
	CSM score	−0.039	0.034	−1.15	0.251	−0.105, 0.027
	Male	−0.328	0.437	−0.75	0.453	−1.185, 0.529
	Education	−0.257	0.319	−0.80	0.421	−0.883, 0.369
	Smoking	0.268	0.456	0.59	0.557	−0.626, 1.162
	Alcohol use	0.404	0.349	1.16	0.247	−0.280, 1.088
	CESD score	−0.009	0.029	−0.30	0.764	−0.067, 0.048
	Physical activity	−0.0001	0.0005	−0.26	0.798	−0.001, 0.0001
	Modified PSQI	0.072	0.078	0.92	0.357	−0.081, 0.225
	Breakfast time	0.057	0.130	0.44	0.659	−0.197, 0.312
	Dinner time	−0.105	0.115	−0.91	0.361	−0.329, 0.120
	Total daily calories	−0.0004	0.0004	−1.17	0.242	−0.001, 0.0003

Mediation analysis in participants aged ≥60 years is shown in Table 6. The BMI outcome model revealed that increased sleep duration was significantly associated with decreased BMI, with a coefficient of −0.283 (95% CI: −0.439, −0.126). More morning preference (higher CSM score) was also significantly associated with lower BMI (*p* = 0.008). Social jetlag, however, was not significantly associated with BMI (*p* = 0.962). A bootstrap with 1,000 replications was then performed (Table 7). The results revealed the ACME_1_ was significant, i.e., that every one unit increase in CSM score (more morning preference) would be associated with an increase in sleep duration, which then significantly decreased BMI by 0.016 kg/m^2^ (95% CI: −0.028, −0.005). However, the association between CSM score and BMI mediated by social jetlag was not significant (ACME_2_ = 0.00007, 95% CI: −0.003, 0.003). In addition, we found that there was also a direct effect of CSM on BMI such that every one unit increase in CSM score (more morning preference) was associated with a decrease in BMI by 0.059 kg/m^2^ (95% CI: −0.106, −0.013).

**Table 6:** Multiple mediation analysis of CSM score and body mass index in participants age >60 years (*n* = 1,358).

**Equations**	**Factor**	**Coefficient**	**SE**	***Z***	***P***	**95% CI**
Sleep duration	CSM score	0.058	0.008	7.74	<0.001	0.043, 0.073
	Male	0.138	0.105	1.31	0.191	−0.068, 0.344
	Education	0.019	0.073	0.26	0.795	−0.125, 0.163
	Smoking	0.053	0.116	0.46	0.645	−0.173, 0.280
	Alcohol use	0.132	0.084	1.57	0.116	−0.033, 0.298
	CESD score	−0.015	0.008	−1.82	0.069	−0.031, 0.001
	Physical activity	−0.0002	0.0001	−1.63	0.103	−0.0005, 0.00004
	Modified PSQI	0.037	0.019	1.93	0.053	−0.0005, 0.074
	Breakfast time	0.061	0.031	1.97	0.049	0.0003, 0.122
	Dinner time	−0.033	0.028	1.19	0.235	−0.089, 0.022
	Total daily calories	3.47*e*−06	0.00009	0.04	0.968	−0.0002, 0.0002
Social jetlag	CSM score	−0.005	0.002	−2.60	0.009	−0.009, −0.001
	Male	−0.015	0.028	−0.51	0.607	−0.070, 0.041
	Education	0.008	0.020	0.41	0.681	−0.030, 0.047
	Smoking	0.039	0.031	1.27	0.204	−0.021, 0.100
	Alcohol use	−0.010	0.023	0.46	0.648	−0.055, 0.034
	CESD score	−0.0002	0.002	−0.07	0.946	−0.005, 0.004
	Physical activity	−0.00002	0.00003	−0.58	0.565	−0.0001, 0.00005
	Modified PSQI	0.0009	0.005	0.18	0.856	−0.009, 0.011
	Breakfast time	0.020	0.008	2.39	0.017	0.004, 0.036
	Dinner time	−0.005	0.008	−0.71	0.475	−0.020, 0.009
	Total daily calories	0.00002	0.00002	0.83	0.405	−0.00003, 0.00006
BMI outcome	Social jetlag	−0.014	0.297	−0.05	0.962	−0.596, 0.568
	Sleep duration	−0.283	0.080	−3.54	<0.001	−0.439, −0.126
	CSM score	−0.059	0.023	−2.63	0.008	−0.104, −0.015
	Male	−0.204	0.309	−0.66	0.509	−0.810, 0.402
	Education	−0.569	0.215	−2.65	0.008	−0.991, −0.147
	Smoking	−0.027	0.340	−0.08	0.937	−0.693, 0.639
	Alcohol use	0.282	0.248	1.14	0.256	−0.204, 0.768
	CESD score	−0.045	0.024	−1.85	0.065	−0.093, 0.002
	Physical activity	−0.0005	0.0004	−1.22	0.222	−0.001, 0.0003
	Modified PSQI	0.057	0.056	1.03	0.304	−0.052, 0.167
	Breakfast time	−0.025	0.092	−0.27	0.787	−0.205, 0.155
	Dinner time	−0.175	0.083	−2.11	0.035	−0.337, −0.012
	Total daily calories	−0.0004	0.0003	−1.67	0.095	−0.0009, 0.0001

**Table 7:** Causal association effects between CSM score and body mass index in participants age >60 years.

**Parameter**	**Model**	**Pathway**	**Beta**	**95% CI**
ACME_1_	Sleep duration model	CSM→Sleep duration→BMI (a_1_b_1_)	−0.016	−0.028, −0.005
ACME_2_	Social jetlag model	CSM→Social jetlag→BMI (a_2_b_2_)	0.00007	−0.003, 0.003
Direct effect	BMI model	CSM→BMI (c′)	−0.059	−0.106, −0.018
Total effects			0.076	0.031, 0.121
**PERCENTAGE OF DIRECT AND MEDIATION EFFECTS OF CSM ON BMI**				
Percent mediation effect through sleep duration			22	
Percent mediation effect through social jetlag			0	
Direct effect			78	

*ACME, average causal mediation effect*.

## Discussion

In this large cohort of patients with prediabetes who were non-shift workers, we demonstrated that more evening preference was independently associated with higher BMI, after adjusting for multiple covariates. This was mainly due to a direct relationship (estimated at 80%) and also was partly mediated by shorter sleep duration (estimated at 16%). This was especially true for our participants who were older than 60 years. In this group, while greater social jetlag was associated with more evening preference, it was neither significantly associated with BMI after adjusting for confounders, nor did it mediate the relationship between morningness-eveningness and BMI. For participants aged ≤60 years, social jetlag was a predominant predictor of BMI while evening preference did not play a significant role. This could possibly be due to the finding that social jetlag was greater in younger group in our study (*p* = < 0.001) which was similar to previous report (14).

In our cohort, a difference in CSM score between 90th and 10th percentile (more evening preference) was associated with an increase in BMI by 0.102 kg/m^2^ mediated through sleep duration, and was directly associated with an increase in BMI by 0.511 kg/m^2^. In those aged ≤60 years, 1 h increase in social jetlag was associated with an increase in BMI by 0.556 kg/m^2^.These effect sizes could be clinically significant, as in the Diabetes Prevention Program, 7% weight loss (approximately a 2.2 kg/m^2^ reduction in BMI in this population with baseline weight of 94.2 kg) by diet and exercise in participants with impaired glucose tolerance resulted in a 58% reduction in the risk of developing diabetes during a follow up of 2.8 years (3). Our results highlight the relationship between circadian preference, social jetlag, sleep duration, and adiposity, further supporting the role of circadian regulation on BMI. These results could inform further interventional studies to reduce BMI in this patient group who are at high risk of developing diabetes. Whether circadian preference and social jetlag represent risk factors for future diabetes development requires further follow up of this cohort.

Mechanisms underlying the relationship between circadian regulation and energy metabolism were elucidated in well-controlled experiments inducing circadian misalignment. After 10 days of forced-desynchrony in 10 healthy participants, leptin levels decreased by 17% and a daily cortisol rhythm reversed (5). In a separate experiment combining sleep restriction and circadian misalignment for 3 weeks, mimicking night shift work, leptin profile was observed to be slightly decreased while ghrelin profile slightly increased, along with an 8% reduction in resting metabolic rate (6). Furthermore, a 6-day inpatient simulated night shift protocol led to a 3% decrease in total energy expenditure as measured by a whole-room calorimeter (24). These data are supported by emerging evidence from population-based studies focusing on the role of evening preference, typically associated with mild form of circadian misalignment, and overweight/obesity. In a study of 511 adolescents, evening types had significantly higher BMI z-scores than morning types (15). Furthermore, evening preference has been shown to be associated with weight gain (25) or failed attempts to lose weight (26, 27). In the National Weight Control Registry, morning chronotype was associated with weight loss maintenance (26). Among 252 severely obese adults undergoing bariatric surgery, those who were evening-type had significantly higher BMI and higher weight regain 4 years after surgery than those who were morning-type (27). The results from the current study are in agreement with these data and provide significant evidence of the relationship between evening preference and BMI in patients with prediabetes, a group which BMI is an important predictor of diabetes development.

Those with more evening preference may have certain behaviors which contribute to the relationship between eveningness and BMI. Evening types were described to be associated with insufficient sleep duration (28, 29), the findings confirmed in our study. This could possibly be due to preferred later sleep timing with the need to wake up earlier than desired to conform to the general society's schedule. The mechanisms linking insufficient sleep and increased obesity risk have been well-characterized in experimental sleep restriction studies, including alterations in appetite regulating hormones (30–32), increased hunger/appetite and unhealthy food consumption (33–35), and little or no change in energy expenditure which could not compensate for increased caloric intake (36–38). The results from these experimental studies are well-supported by epidemiological studies linking short sleep to obesity (39). In a meta-analysis of over 600,000 adults, each hour of shorter sleep duration was associated with 0.35 kg/m^2^ change in BMI (39). Insufficient sleep may also hinder the effectiveness of weight loss. In an experiment involving 10 overweight adults for 14 days, those sleeping 8.5 h had a greater loss of fat-free body mass than those assigned to 5.5 h time in bed (40). Emerging data suggested that adequate sleep may be beneficial in weight loss. In a study of 10 overweight adults with short habitual sleep duration (<6.5 h), home sleep extension for 2 weeks (by 1.6 h) was associate with a 14% decrease in overall appetite and a 62% decrease in desire for sweet and salty foods (41). In another study of 123 obese/overweight individuals who underwent a low calorie diet intervention for 14–24 weeks, longer self-reported sleep duration and better sleep quality were associated with greater fat mass loss measured by a dual-energy x-ray absorptiometry (42). A recent randomized study explored the effects of caloric restriction with or without sleep restriction for 8 weeks (43). While both groups lost similar amount of weight, sleep restriction group (average sleep reduction of 169 min/week) lost significantly less proportion of total mass lost as fat (43). Our data, derived exclusively from prediabetes patients, supported these previous findings and suggested that sleep extension should be explored as an adjunct to diet and exercise in reducing diabetes risk.

Besides sleep duration, other behaviors associated with more evening preference could be contributing to increased BMI. Greater social jetlag, often seen in those with more evening preference, was described to be associated with higher BMI in those with baseline BMI ≥ 25 kg/m^2^ in a large population based study of more than 60,000 individuals in Europe (14). Our study, with participants' mean BMI of 25.8 kg/m^2^, revealed that social jetlag, a marker of circadian misalignment, was associated with BMI in those aged ≤60 years. This was likely due to greater social jetlag in younger age group. This result was in agreement with a recent study of participants with non-communicable chronic diseases (mean age 55 years) which revealed the association between social jetlag and being overweight (44). In older participants, despite the association between social jetlag and evening preference, social jetlag was not an independent predictor of BMI after adjusting for other covariates, thus other factors could play a role. Meal timing, an important input of the circadian system, could be a factor, as delayed or mistimed meals could lead to alterations and uncoupling between the central and peripheral oscillators (10, 45). Consuming food at a later time of the day was shown to be associated with higher BMI (46), development of obesity (47) and less weight loss in response to weight loss therapy (48, 49). In our cohort, however, later dinner time was associated with lower BMI. This could possibly be due to recall bias of the dietary information, or that the relationship between meal timing and individual circadian timing is more important than the meal time as expressed by clock time itself. This was supported by a recent study in 110 young adults which found that consumption of food during the circadian evening (as assessed by dim-light melatonin onset) was associated with higher BMI while the clock hour of food consumption had no effects (50). Besides dinner time, breakfast is another important factor as evening types were associated with breakfast skipping or morning anorexia (51, 52). Less caloric consumption at breakfast or breakfast skipping were shown to be related to increased adiposity (53, 54) and sleep-wake irregularity (55).

In addition to social jetlag and meal timing, evening types generally prefer later sleep timing. This could be associated with greater exposure of light at night leading to circadian disruption. Experimental exposure to blue light 2 h before bedtime in young men led to decreased energy expenditure during the following morning (56). Population based studies have also supported the association between light at night and overweight/obesity (57, 58). Sleep time in our cohort, while correlated with CSM, was not an independent predictor of BMI (data not shown) but the information on light exposure at night was not available. Lastly, more evening preference has been shown to be associated with unhealthy diet, which could contribute to obesity in a long term (15, 59). It is likely that a combination of these factors, rather than any alone, contributes to overweight/ obesity in those with more evening preference. Whether comprehensively targeting these behaviors associated with more evening preference will reduce BMI and possibly future diabetes risk, and the contribution of each factor, is a subject of future research.

Given that the study was performed only in Thai population, some differences from other population should be noted. According to the CSM cutoff, our population mostly had morning preference (a cutoff of 44) (60). It is known that geographic location, likely due to temperature and sun light exposure, is related to circadian preference with countries closer to the equator being more morning (61, 62). Alternatively, a cutoff based on population has been suggested using 10th and 90th percentile for evening and morning preference, respectively (60). Even though our population had relatively more morning preference, we did see a relationship between CSM and BMI across CSM continuum. Another aspect which could be culturally related is meal timing. Breakfast and dinner timing in this cohort are typical of our population which differ from some others such as Spain which typical lunch time is 3 p.m. (48) or in the United States that eating intervals often extend beyond 15 h (63). These differences could play a role our results on relationship between meal timing and BMI.

Our study has the strengths of enrolling a large number of participants with prediabetes, along with comprehensive assessments of circadian preference, sleep and dietary intake. However, there are limitations. The study was conducted at one medical center in Thailand, and thus may not reflect findings in a general population. Sleep assessments, although obtained through validated questionnaires, were subjective. Dietary recall is subject to imprecision such as participants' ability to recall their food intake and timing, and a tendency to underreport which is a well-known phenomenon (64). An average of only 1,027 calories per day could be possibly related to the attempt of the participants to limit their food intake prior to their doctor's visit when most of the assessments occurred. However, excluding the caloric information did not alter the results of our analyses. In addition, the information on light exposure, especially at night, is not available in this study.

In summary, in patients with prediabetes, more evening preference was directly associated with higher BMI and indirectly through insufficient sleep duration. These data supported the importance of circadian regulation in energy metabolism and could inform further interventional studies to reduce BMI in this high risk group.

## Author contributions

TA planned the study, collected and analyzed the data, wrote and edited the manuscript. DL and ST collected the data and reviewed/edited the manuscript. AT planned the study, analyzed the data, and reviewed/edited the manuscript. SR planned the study, contributed to discussion, wrote and edited the manuscript.

### Conflict of interest statement

SR reports grants from Merck Sharp and Dohme, non-financial support from ResMed, personal fees from Novo Nordisk, personal fees from Sanofi Aventis, personal fees from Medtronic, outside the submitted work. The remaining authors declare that the research was conducted in the absence of any commercial or financial relationships that could be construed as a potential conflict of interest.
